# Supplementary material for: Thermodynamic sampling of materials using neutral-atom quantum computers
Source: npj Quantum Inf. 2026 Jul 21;12(1):183. doi: 10.1038/s41534-026-01323-x (PMC13751300; doi:10.1038/s41534-026-01323-x)
Supplement: Supplementary file 1 [file 41534_2026_1323_MOESM1_ESM.pdf]

Supplementary Information: Thermodynamic sampling of materials using neutral-atom quantum computers

Bruno Camino[1*], Mao Lin[2*], John Buckeridge[3] and Scott M. Woodley[1]

[1*]Chemistry Department, University College London, 20 Gordon St, London, WC1H 0AJ, United Kingdom.
[2]Amazon Braket, Seattle, 98170, WA, United States.
[3]School of Engineering, London South Bank University, 103 Borough Rd, London, SE10 AA, United Kingdom.

*Corresponding author(s). E-mail(s): b.camino@ucl.ac.uk; maolinml@amazon.com;
Contributing authors: j.buckeridge@lsbu.ac.uk; scott.woodley@ucl.ac.uk;

Dataset construction and nitrogen concentration distribution

The training and test sets used to fit and validate the Rydberg Hamiltonian are constructed in a two-stage process. First, nitrogen-doped configurations are generated at random within the 78-site graphene supercell, enforcing that no two configurations in the pool are related by the symmetry operations of the pristine lattice. These symmetry-independent configurations (SICs) represent the independent information content of the dataset: a DFT geometry optimisation and energy evaluation is performed for each SIC.

In the second stage, the space-group operations of the graphene supercell are applied to each SIC to generate all symmetry-equivalent configurations (SECs). Every SEC inherits the DFT energy of its parent SIC; no additional DFT calculations are performed. The SECs are included in the linear regression (Eq. 1 of the main text) so that all symmetry-equivalent sites receive the same fitted parameters in the Rydberg Hamiltonian.

The resulting dataset comprises 28 SICs each for training and test, spanning nitrogen concentrations from 1 to 10 atoms per 78-site cell (1.3%–12.8%). Supplementary Table S1 reports the number of SICs and the corresponding number of SECs as a function of nitrogen concentration. After symmetry expansion, the training and test sets contain 2145 and 2184 configurations, respectively. We stress that these totals reflect symmetry multiplicities, not independent DFT calculations.

Table S1 Number of symmetry-independent configurations (SICs) and symmetry-equivalent configurations (SECs) as a function of the number of nitrogen atoms per 78-site supercell, for both training and test sets.

N atoms	Train SICs	Test SICs	Train SECs	Test SECs
1	1	1	78	78
2	3	3	195	234
3	3	3	234	234
4	3	3	234	234
5	3	3	234	234
6	3	3	234	234
7	3	3	234	234
8	3	3	234	234
9	3	3	234	234
10	3	3	234	234
Total	28	28	2145	2184

Convergence with respect to training set size

To assess whether the linear regression is converged with respect to the number of training configurations, we repeated the fit using random subsets of the training SICs at 25%, 50%, 75%, and 100% of the full training set (7, 14, 21, and 28 SICs, respectively). For each sub-100% fraction, ten independent random draws were performed and the root-mean-square error (RMSE) was evaluated on the full test set in every case.

Supplementary Figure S1 shows the resulting train and test RMSE as a function of the number of training SICs. The test RMSE decreases from 4.10×10^{-4} eV/atom at 25% to 3.13×10^{-4} eV/atom at 100%, with the largest improvement occurring between 25% and 50%. Beyond 50% of the training data (14 SICs), the test RMSE is effectively constant, indicating that the regression is well converged with the 28 SICs used in the main text. The full model achieves $R^2 = 0.96$ on the training set and a Spearman rank correlation of $\rho = 0.986$ on the test set.

Convergence with respect to interaction cutoff

The two-body interaction in the Rydberg Hamiltonian decays as R^{-6}, and the regression in the main text is truncated at the fourth-nearest-neighbour (4NN) shell. To

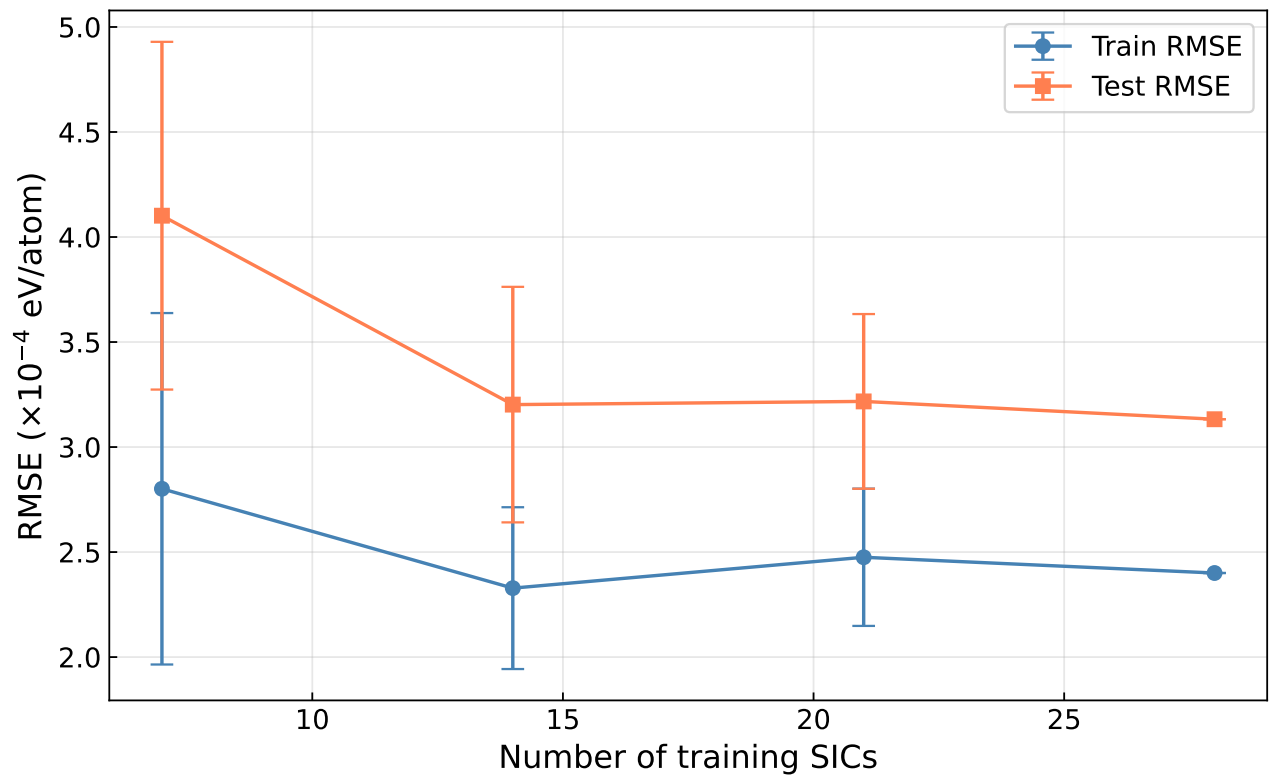

Fig. S1 Train and test RMSE as a function of the number of training SICs. Error bars for sub-100% fractions indicate the standard deviation over ten independent random subsamples.

verify that this truncation is sufficient, we fitted the model at each cutoff from 1NN to 5NN using the full training set and evaluated on the full test set. The results are plotted in Supplementary Figure S2.

The only significant change occurs between 1NN and 2NN, where the inclusion of the next-nearest-neighbour shell reduces the test RMSE by approximately 2.5%. Beyond 2NN, both the RMSE and the fitted coefficients are essentially unchanged: the values at 3NN, 4NN, and 5NN agree to within 0.2%. The 4NN cutoff adopted in the main text is therefore a conservative choice, well within the converged regime.

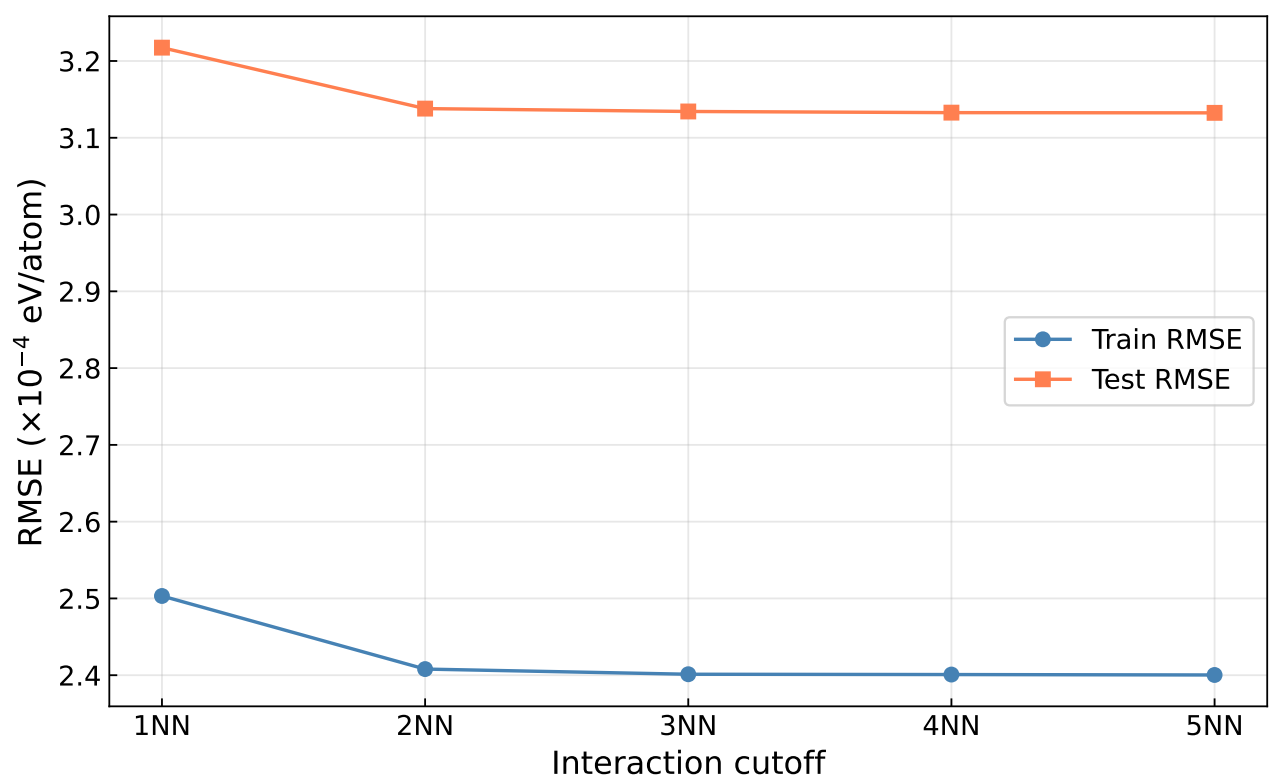

Fig. S2 Train and test RMSE as a function of the interaction cutoff, from first (1NN) to fifth (5NN) nearest neighbours.
